# Supplementary material for: Controlling opioid receptor functional selectivity by targeting distinct subpockets of the orthosteric site
Source: eLife. 2021 Feb 8;10:e56519. doi: 10.7554/eLife.56519 (PMC7909954; doi:10.7554/eLife.56519)
Supplement: Transparent reporting form [file elife-56519-transrepform.docx]

***eLife’s* transparent reporting form**

We encourage authors to provide detailed information *within their submission* to facilitate the interpretation and replication of experiments. Authors can upload supporting documentation to indicate the use of appropriate reporting guidelines for health-related research (see [EQUATOR Network](http://www.equator-network.org/%20)), life science research (see the [BioSharing Information Resource](https://biosharing.org/)), or the [ARRIVE guidelines](http://www.plosbiology.org/article/info:doi/10.1371/journal.pbio.1000412) for reporting work involving animal research. Where applicable, authors should refer to any relevant reporting standards documents in this form.

If you have any questions, please consult our Journal Policies and/or contact us: [editorial@elifesciences.org](mailto:editorial@elifesciences.org).

**Sample-size estimation**

- You should state whether an appropriate sample size was computed when the study was being designed
- You should state the statistical method of sample size computation and any required assumptions
- If no explicit power analysis was used, you should describe how you decided what sample (replicate) size (number) to use

Please outline where this information can be found within the submission (e.g., sections or figure legends), or explain why this information doesn’t apply to your submission:

Number of technical replicates and biological replicates are reported in the figure legends or methods or supplementary section of the manuscript. Sample size was determined based on variability of the response deviating from the mean as indicated by the standard error of the mean (SEM), which is also represented in the figures.

Typically, at least three biological replicates were performed so that the SEM was within at least 20% of the mean, but exact number of

replicates are indicated per result in figure legends or methods section or supplementary.

For Pharmacological/Behavioral studies: All experiments were performed in two or three separate, independent cohorts (replicates), with total results analyzed together. On the basis of analysis aiming for 95% power with an alpha significance of p=0.05 and an ANOVA analysis with repeated measures within-between interaction, G*Power 3.19.2 was used to calculate in advance of testing a minimum number of mice needed per experiment.  Under these criteria:

Antinociceptive testing of agonist properties: with an assumed moderate effect size (f=1.0) and two measurements assumed across 5 groups, an estimated sample size of 8 mice per analgesic testing condition will be needed, with a predicted critical F-value of 6.608 and actual power = 0.998.

For CLAMS testing (respiratory or locomotor studies): the assumed small effect size (f=0.5) with 32 measurements (every 5 min across 180 min) produces an estimated sample size of 10 mice per testing condition, with a predicted F-value of 1.321 and actual power of 0.999. Note that a subset of these conditions were replicated (n=20) to verify initial findings. Additional data did not change the interpreted results, so all data is included in this report.

Assuming a small effect size (f=0.5) and two measures, G*Power estimated 24 mice per condition for conditioned place preference will be needed to achieve statistical significance in this work, defined with a critical F-value of 3.098 and with actual power = 0.973.

Notably, experience and a review of related literature shows that all these values are consistent with published results, given anticipated variance.

Note that for each cohort of data, use of ANOVA with appropriate post hoc test and Student’s t-tests of statistical significance were used to minimize the number of animals required. A value of p<0.05 was determined to be adequate to determine significance, and no additional animals will be used. This criteria was used to reduce the number of mice used in place-conditioning testing, consistent with both ARRIVE guidelines and NIH’s “three Rs” guidance for animal testing.

**Replicates**

- You should report how often each experiment was performed
- You should include a definition of biological versus technical replication
- The data obtained should be provided and sufficient information should be provided to indicate the number of independent biological and/or technical replicates
- If you encountered any outliers, you should describe how these were handled
- Criteria for exclusion/inclusion of data should be clearly stated
- High-throughput sequence data should be uploaded before submission, with a private link for reviewers provided (these are available from both GEO and ArrayExpress)

Please outline where this information can be found within the submission (e.g., sections or figure legends), or explain why this information doesn’t apply to your submission:

Data were replicated using technical and independent replicates. See figure legends or methods or supplementary section for specific details. Typically, at least three biological replicates were performed. No data were excluded for this study.

**Statistical reporting**

- Statistical analysis methods should be described and justified
- Raw data should be presented in figures whenever informative to do so (typically when N per group is less than 10)
- For each experiment, you should identify the statistical tests used, exact values of N, definitions of center, methods of multiple test correction, and dispersion and precision measures (e.g., mean, median, SD, SEM, confidence intervals; and, for the major substantive results, a measure of effect size (e.g., Pearson's r, Cohen's d)
- Report exact p-values wherever possible alongside the summary statistics and 95% confidence intervals. These should be reported for all key questions and not only when the p-value is less than 0.05.

Please outline where this information can be found within the submission (e.g., sections or figure legends), or explain why this information doesn’t apply to your submission:

Data were analyzed using Prism (8.1.1, GraphPad) as stated in the Materials and Methods section. Error bar definition and statistical test are indicated in the figure legends and materials and methods section.

(For large datasets, or papers with a very large number of statistical tests, you may upload a single table file with tests, Ns, etc., with reference to sections in the manuscript.)

**Group allocation**

- Indicate how samples were allocated into experimental groups (in the case of clinical studies, please specify allocation to treatment method); if randomization was used, please also state if restricted randomization was applied
- Indicate if masking was used during group allocation, data collection and/or data analysis

Please outline where this information can be found within the submission (e.g., sections or figure legends), or explain why this information doesn’t apply to your submission:

No data required randomization

**Additional data files (“source data”)**

- We encourage you to upload relevant additional data files, such as numerical data that are represented as a graph in a figure, or as a summary table
- Where provided, these should be in the most useful format, and they can be uploaded as “Source data” files linked to a main figure or table
- Include model definition files including the full list of parameters used
- Include code used for data analysis (e.g., R, MatLab)
- Avoid stating that data files are “available upon request”

Please indicate the figures or tables for which source data files have been provided:

n/a
